# The relationship between circulating 25-hydroxyvitamin D and survival in newly diagnosed advanced non-small-cell lung cancer

**DOI:** 10.1186/s12885-015-2043-x

**Published:** 2015-12-24

**Authors:** Pankaj G. Vashi, Persis Edwin, Brenten Popiel, Digant Gupta

**Affiliations:** Cancer Treatment Centers of America® (CTCA) at Midwestern Regional Medical Center, 2520 Elisha Ave, Zion, IL 60099 USA

**Keywords:** Serum 25-hydroxyvitamin D, Lung cancer, Survival

## Abstract

**Background:**

Serum 25-hydroxyvitamin D [25(OH)D], the major circulating form of vitamin D used for evaluating the vitamin D status of patients, has been associated with survival in a variety of cancers with conflicting evidence. We aimed to investigate this association in newly diagnosed advanced non-small-cell lung cancer (NSCLC) patients.

**Methods:**

This was a consecutive cohort of 359 newly diagnosed stages III-IV NSCLC patients who underwent a baseline serum 25(OH)D evaluation prior to receiving any treatment at our institution between January 2008 and December 2010. We used the vitamin D categories of “deficient (<20 ng/ml)” and “not deficient (> = 20 ng/ml)”. Cox regression was used to evaluate the prognostic significance of serum 25(OH)D after adjusting for relevant confounders.

**Results:**

Mean age at diagnosis was 57.4 years. Of the 359 patients, 151 (42.1 %) were deficient in vitamin D at the time of diagnosis. The median survival in deficient and not deficient cohorts was 11.7 and 12.8 months respectively (*p* = 0.06). Season of diagnosis, performance status, smoking status and hospital location significantly predicted vitamin D status. On univariate Cox analysis, gender, stage of disease, hospital location, histologic subtype, subjective global assessment (SGA), performance status, smoking status, body mass index and serum albumin were significantly associated with survival (*p* <0.05 for all). On multivariate Cox analysis, six variables demonstrated statistically significant associations with survival: stage of disease, hospital location, histologic subtype, SGA, smoking status and serum albumin (*p* <0.05 for all). Serum vitamin D, which was borderline significant in univariate analysis, lost its significance in multivariate analysis.

**Conclusions:**

We found season of diagnosis, performance status and smoking history to be predictive of vitamin D status. Consistent with previously published research in advanced NSCLC, we did not find any significant association between pre-treatment serum 25(OH)D and survival in our patients.

## Background

Vitamin D produced in the skin upon sun exposure or ingested from the diet is converted in the liver to 25-hydroxyvitamin D [25(OH)D], the major circulating form of vitamin D used for evaluating the vitamin D status of patients [1, 2]. 25(OH)D is hydroxylated in the kidneys to form the biologically active metabolite 1,25-dihydroxyvitamin D [1,25(OH)_2_D] [3, 4]. Though 25(OH)D is not the active form of vitamin D, it is known to be the best indicator of vitamin D status as it accurately reflects vitamin D intake from all sources and has a half-life of two to three weeks compared to only four hours for the active form (1,25(OH)_2_D) [5].

Emerging evidence in the literature suggests an association between serum 25(OH)D and survival in several types of cancer, however, the evidence is not conclusive with regard to the direction and strength of association. While several studies have demonstrated a positive association between serum vitamin D and survival in multiple cancer types including gastric [6], colorectal [7–11], breast [12, 13] and prostate [14], other studies have demonstrated a lack of such an association [15–17]. Some studies have combined newly diagnosed and previously treated patients in the same analysis [14], while others have found the vitamin D-survival association to become attenuated after adjusting for important confounders [15]. Collectively, these studies indicate that using a homogeneous patient population and adjustment of important confounders are important aspects of study design and data analysis respectively that should be taken into account.

Specific to non-small cell lung cancer (NSCLC), there have been 7 published studies evaluating the relationship between serum 25(OH)D and survival with 3 of them demonstrating positive association, 3 null association and 1 negative association. A study by Zhou et al. conducted in 447 patients with early-stage NSCLC, found higher levels of vitamin D to be associated with improved survival particularly among stage IB-IIB patients [18]. A Norwegian study of 210 lung cancer patients that collected serum samples shortly after diagnosis, observed that higher serum 25(OH)D was associated with a statistically significant longer survival time [19]. A study conducted in 16,693 men and women participating in the Third National Health and Nutrition Examination Survey found serum 25(OH)D concentrations to be inversely associated with lung cancer mortality in nonsmokers; this association was diminished among those with excess circulating vitamin A [20]. Heist et al. conducted a study in 294 patients with stage III-IV NSCLC and found no difference in survival by circulating vitamin D level quartiles [21]. A prospective study by Turner et al. conducted in a relatively homogeneous group of 148 surgically treated lung cancer patients, found that pre-surgical levels of serum 25(OH)D were not associated with either overall or lung-cancer specific mortality, although the study did report a protective effect of higher vitamin D binding protein on lung-cancer specific mortality [22]. The most recent findings on the lack of a significant relationship between serum vitamin D and survival comes from Anic et al. who investigated 500 male lung cancer cases (staged I–IV) in the Alpha-Tocopherol, Beta-Carotene Cancer Prevention Study. Comparing highest to lowest quartiles, there was no statistically significant association between serum 25(OH)D and lung cancer survival [23]. Finally, a Chinese study of 87 NSCLC cases reported a negative association such that higher serum 25(OH)D at diagnosis was associated with a shorter survival time [24]. 

Given this variability in findings on the relationship between serum vitamin D and survival in NSCLC, additional studies with large sample sizes are needed to better understand the direction and strength of this association. We investigated this association in a large homogenous group of newly diagnosed advanced NSCLC patients treated at a national network of oncology hospitals.

## Methods

### Study population

A consecutive series of 359 newly diagnosed stages III–IV NSCLC patients treated at three Cancer Treatment Centers of America® (CTCA) hospitals (located in Zion, IL, Philadelphia, PA, and Tulsa, OK) between January 2008 and December 2010 was evaluated. We included a consecutive case series of patients to minimize the probability of selection bias. The present study was conducted according to the guidelines laid down in the Declaration of Helsinki and was approved by the Midwestern Regional Medical Center Institutional Review Board (IRB) at Cancer Treatment Centers of America®. The IRB waived the need for informed consent because there was no direct patient contact in this study. This study involved collection of existing data from patient records in such a manner that subjects could not be identified, directly or through identifiers linked to the subjects.

### Vitamin D measurement

Serum samples were collected within 30 days of first visit at our hospital, and prior to initiation of anticancer therapy. Serum was collected at the laboratory, packed in coolpacks and sent to the Laboratory Corporation of America (Raleigh, NC) where a chemiluminescence immune assay (CLIA, DiaSorin Liasion assay) was used to measure 25(OH)D. Serum samples were incubated with antivitamin-D coated microparticles and isoluminol derivative-conjugated 25(OH)D before measurement of chemiluminescent signals. Analysis was completed within 48 h of collection. The DiaSorin Liasion 25(OH)D assay has been clinically validated to be comparable in accuracy and precision to the radioimmunoassay (RIA). This method uses the same particles used in the DiaSorin RIA technique. Studies have found this to be a rapid, accurate, and precise tool for the measurement of serum 25(OH)D [25, 26].

### Statistical analysis

Serum 25(OH)D was the primary independent variable of interest. We used the categories “deficient (<20 ng/ml)”, and “not deficient (> = 20 ng/ml)” in accordance with previously published research in this area [12, 14]. A comparison of clinical and demographic characteristics was made between the two vitamin D categories using a two-sample t-test, a Mann Whitney test or a chi-square test depending upon the underlying distribution of the variables.

The primary endpoint was patient survival and was defined as the time interval between the date of first serum vitamin D assessment and the date of death from any cause or the date of last contact/last known to be alive. Patients were followed prospectively until December 2014. The Kaplan-Meier method was used to calculate survival. The log rank test statistic was used to evaluate the equality of survival distributions across the 2 serum 25(OH)D groups. Clinical, demographic and serum 25(OH)D variables were evaluated using univariate Cox proportional hazards models to determine which parameters showed individual prognostic value for survival. Multivariate Cox proportional hazards models were then performed to evaluate the independent prognostic significance of all variables that were evaluated in univariate analysis. We adjusted for the following variables in the multivariate analysis: age, gender, CTCA hospital, stage of disease, Eastern Cooperative Oncology Group (ECOG) performance status, body mass index (BMI), season of diagnosis, serum albumin, smoking status, histologic subtype, and nutritional status as measured using Subjective Global Assessment (SGA). Season of diagnosis was defined as winter: December-February; spring: March-May; summer: June-August, or fall: September-November. The effect of serum 25(OH)D and other variables on patient survival was expressed as hazard ratios (HRs) with 95 % confidence intervals (CIs).

Cox regression with time-invariant covariates assumes that the ratio of hazards for any two groups remains constant in proportion over time. We checked this proportional hazards (PH) assumption using a combination of graphical and statistical testing procedures. First, we examined log-minus-log plots for categorical predictors. As a second approach, we ran an extended Cox model with time-dependent covariates for continuous predictors. Finally, a goodness-of-fit testing approach based on Schoenfeld residuals was used to evaluate the PH assumption.

Finally, to assess the possible influence of sample bias on the results, as well as to investigate the stability of the model coefficients, we performed a bias-corrected and accelerated (BCa) bootstrap resampling procedure. We generated 1000 samples, each the same size as the original data set, by random selection with replacement. Cox regression was then run separately on these 1000 samples to obtain robust estimates of the standard errors of coefficients, and hence the p values and 95 % BCa CIs of the model coefficients [27].

No formal sample size calculations were conducted for this analysis. All reported P values are from two-sided tests. All statistical analyses utilized SPSS version 20.0 (International Business Machines, Armonk, New York, USA).

## Results

### Patient characteristics

Table 1 displays the baseline characteristics of our patients. The median follow-up was 10.8 months. At the time of this analysis, 293 (81.6 %) patients had expired while 66 (18.4 %) were considered censored. 163 (45.4 %) patients were taking vitamin D supplements at the time of diagnosis. 332 (92.5 %) patients received chemotherapy, 191 (53.2 %) received radiation therapy and 24 (6.7 %) received surgery at our institution. A total of 172 (47.9 %) patients received both chemotherapy and radiation therapy at our institution.

**Table 1:** Patient characteristics for the overall population as well as stratified by 2 serum vitamin D categories

Categorical variables	Overall population (*n* = 359)	Deficient: <20 ng/ml (*n* = 151)	Not deficient: > = 20 ng/ml (*n* = 208)	*P*-value
Gender				0.85
Male	181	77 (42.5)	104 (57.5)	
Female	178	74 (41.6)	104 (58.4)	
Stage				0.90
III	94	39 (41.5)	55 (58.5)	
IV	265	112 (42.3)	153 (57.7)	
CTCA hospital				0.04*
Philadelphia, PA	50	13 (26)	37 (74)	
Zion, IL	269	119 (44.2)	150 (55.8)	
Tulsa, OK	40	19 (47.5)	21 (52.5)	
Histologic subtype^a^				0.90
Adenocarcinoma	268	114 (42.5)	154 (57.5)	
Squamous cell	67	28 (41.8)	39 (58.2)	
Others	19	9 (47.4)	10 (52.6)	
SGA^a^				0.07
Well-nourished	211	81 (38.4)	130 (61.6)	
Moderately-severely malnourished	124	60 (48.4)	64 (51.6)	
ECOG score^a^				0.02*
0–1	233	88 (37.8)	145 (62.2)	
2–4	102	52 (51)	50 (49)	
Season of diagnosis				0.004*
Summer	93	34 (36.6)	59 (63.4)	
Fall	90	27 (30)	63 (70)	
Winter	72	34 (47.2)	38 (52.8)	
Spring	104	56 (53.8)	48 (46.2)	
Smoking Status^a^				0.006*
Never	69	27 (39.1)	42 (60.9)	
Former	143	49 (34.3)	94 (65.7)	
Current	143	75 (52.4)	68 (47.6)	
Vitamin D supplementation at diagnosis				0.92
Yes	163	69 (42.3)	94 (57.7)	
No	196	82 (41.8)	114 (58.2)	
Continuous variables	Overall population (*n* = 359)	Deficient: <20 ng/ml (*n* = 151)	Not deficient: > = 20 ng/ml (*n* = 208)	*P*-value
Mean (SD) age (years)	57.4 (8.4)	57.1 (7.4)	57.6 (9.0)	0.62
Mean (SD) vitamin D (ng/ml)	25.5 (15.3)	13.3 (4.1)	34.4 (14.3)	<0.001*
Mean (SD) BMI (kg/m2)	25.8 (5.7)	25.5 (5.9)	26 (5.7)	0.43
Mean (SD) serum albumin (g/dl)	3.6 (0.57)	3.5 (0.57)	3.6 (0.57)	0.06

(*SGA* Subjective Global Assessment, *ECOG* Eastern Cooperative Oncology Group, *CTCA* Cancer Treatment Centers of America, *PA* Pennsylvania, *IL* Illinois, *OK* Oklahoma, *BMI* Body Mass Index, *g/dl* grams per deciliter, *ng/ml* nanograms per milliliter, *kg/m2* kilograms per meter squared, *SD* Standard Deviation)

*P < = 0.05, Values in parentheses are row percentages

^a^Missing data (Histology = 5; SGA = 24; ECOG = 24; Smoking status = 4)

### Predictors of vitamin D status

Table 1 also describes the patient characteristics stratified by the 2 categories of serum vitamin D (deficient and not deficient). Season of diagnosis, ECOG performance status, smoking status and CTCA hospital were the four variables that demonstrated statistical significance (*p* <0.05). Patients who presented to us in the summer and fall months were less likely to be deficient in vitamin D compared to those who presented in winter and spring. The mean (standard deviation [sd]) serum vitamin D levels were 24.7 (11.8), 31.2 (20.8), 22.3 (10.4) and 23.5 (14.1) nanograms per milliliter (ng/ml) for summer, fall, winter and spring months respectively. Patients with ECOG performance scores of 0–1 were less likely to be deficient in vitamin D compared to those with scores 2–4. Current smoking status was associated with a greater prevalence of vitamin D deficiency compared to past or no smoking history. Finally, patients diagnosed at our Philadelphia hospital had a lower prevalence of vitamin D deficiency compared to patients diagnosed at Zion and Tulsa hospitals. In addition, patients with vitamin D deficiency had lower serum albumin levels compared to those with non-deficient serum vitamin D levels, the finding being borderline significant (*p* = 0.06). Similarly, well-nourished patients had a lower prevalence of vitamin D deficiency compared to malnourished patients, the finding being borderline significant (*p* = 0.07).

### Median survival

**Fig. 1:**
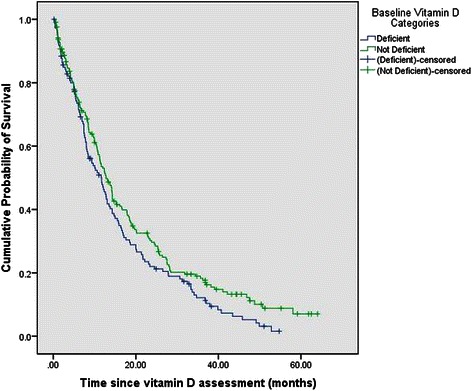
Overall survival stratified by baseline serum 25(OH)D categories. There was no statistically significant difference in the median survival times across the 2 categories of serum vitamin D

Table 2 shows the median survival times as a function of various clinical and demographic variables. There was no statistically significant difference in the Kaplan-Meier median survival times across the 2 categories of serum vitamin D, as displayed in Fig. 1. Gender, stage of disease, CTCA hospital, histologic subtype, SGA, ECOG performance status and smoking status were significantly associated with survival. Females, patients with stage III disease, well-nourished patients, patients with ECOG score 0–1 and patients with no smoking history had a significantly greater median survival compared to males, patients with stage IV disease, patients with ECOG score 2–4, and smoking history respectively. Patients with adenocarcinoma and squamous cell carcinoma had a significantly greater median survival compared to those with cancers in the “others” category. Finally, patients treated at our Philadelphia hospital had a significantly greater median survival compared to those treated at Zion and Tulsa hospitals.

**Table 2:** Median survival as a function of patient characteristics

Categorical variables	Median survival in months	95 % CI	*P*-value
Serum vitamin D			0.06
> = 20 ng/ml	12.8	10.8–14.7	
<20 ng/ml	11.7	8.6–14.7	
Gender			0.04*
Male	11.7	10.2–13.2	
Female	13.5	11.2–15.8	
Stage			<0.001*
III	20.1	12.9–27.3	
IV	10.8	9.2–12.4	
CTCA hospital			<0.001*
Philadelphia, PA	38.2	4.1–72.3	
Zion, IL	12.4	10.6–14.1	
Tulsa, OK	7.4	4.2–10.5	
Histologic subtype			0.002*
Adenocarcinoma	12.8	11.2–14.4	
Squamous cell	11.8	7.7–15.9	
Others	7.2	3.7–10.7	
SGA			<0.001*
Well–nourished	15.2	12.1–18.3	
Moderately-severely malnourished	8.0	6.2–9.9	
ECOG score			<0.001*
0–1	14.3	12.1–16.5	
2–4	8.0	6.2–9.8	
Season of diagnosis			0.73
Summer	11.6	8.4–14.8	
Fall	11.7	8.3–15.1	
Winter	12.6	8.9–16.3	
Spring	12.7	10.2–15.3	
Smoking status			0.02*
Never	19.4	10.8–27.9	
Former	11.0	9.4–12.6	
Current	11.7	9.3–14.1	
Vitamin D supplementation at diagnosis			0.27
Yes	13.6	12.4–14.9	
No	10.6	8.9–12.5	

(*SGA* Subjective Global Assessment, *ECOG* Eastern Cooperative Oncology Group, *CTCA* Cancer Treatment Centers of America, *PA* Pennsylvania, *IL* Illinois, *OK* Oklahoma, *ng/ml* nanograms per milliliter, *CI* Confidence Interval)

*P < = 0.05

To further understand the differences in survival across the 3 hospitals, we evaluated the distribution of baseline clinical and demographic characteristics stratified by the hospital (detailed results not shown in the interest of space). The Philadelphia cohort (66 %) had a significantly greater proportion of females compared to the Zion (47.6 %) and Tulsa (42.5 %) cohorts; *p* = 0.04. The Philadelphia cohort (66.7 %) also had a significantly greater proportion of well-nourished patients compared to the Tulsa cohort (45 %); *p* = 0.04. Further, the Philadelphia cohort (24 %) had a significantly smaller proportion of current smokers compared to the Zion (42.3 %) and Tulsa (47.5 %) cohorts; *p* = 0.002. Finally, the mean baseline serum albumin in the Philadelphia cohort (4.1 grams per deciliter [g/dl]) was significantly higher compared to the Zion (3.4 g/dl) and Tulsa (3.6 g/dl) cohorts; *p* <0.001. There were no systematic differences among the 3 hospitals with regard to age, stage at diagnosis, BMI and ECOG performance status.

The systematic differences among the 3 hospitals with regard to gender, nutritional status, smoking status and serum albumin might, in part, explain the observed differences in median survival.

### Univariate and multivariate survival analysis

Table 3 summarizes the results of univariate and multivariate Cox regression analyses. In the univariate analysis, each predictor was tested in isolation for its association with survival. Gender, stage of disease, CTCA hospital, histologic subtype, SGA, ECOG performance status, smoking status, BMI and serum albumin were significantly associated with survival. Every 1 kilograms per meter squared (kg/m2) increase in BMI was associated with a 3 % reduction in mortality hazard (HR = 0.97; *p* = 0.008) and every 1 g/dl increase in serum albumin was associated with a 56 % reduction in mortality hazard (HR = 0.44; *p* <0.001). In the full model, all variables tested in the univariate analysis were evaluated simultaneously in the same model. Six variables demonstrated statistically significant associations with survival: stage of disease, CTCA hospital, histologic subtype, SGA, smoking status and serum albumin. Every 1 g/dl increase in serum albumin was associated with a 54 % reduction in mortality hazard (HR = 0.46; *p* < 0.001). In the final model, only those 6 variables that were statistically significant in the full model were evaluated together. All of them excepting histologic subtype were found to be statistically significant.

**Table 3:** Univariate and multivariate Cox regression analyses of the relationship between serum vitamin D and survival

Variables	Univariate model HR (95 % CI)	Full model HR (95 % CI)	Final model HR (95 % CI)
Serum vitamin D			
> = 20 ng/ml (reference)			
<20 ng/ml	1.2 (0.9–1.6)	0.99 (0.8–1.3)	
Gender			
Female (reference)			
Male	1.3 (1.01–1.6)*	1.1 (0.9–1.5)	
Stage			
III (reference)			
IV	2.3 (1.8–3.1)*	2.3 (1.7–3.2)*	2.3 (1.7–3.1)*
CTCA hospital			
Philadelphia, PA (reference)			
Zion, IL	3.2 (1.8–5.8)*	1.9 (0.9–4.1)	2.0 (0.9–4.2)
Tulsa, OK	5.8 (3.0–11.2)*	4.0 (1.8–8.8)*	4.1 (1.9–8.9)*
Histologic subtype			
Adenocarcinoma (reference)			
Squamous cell	1.1 (0.8–1.4)	0.9 (0.6–1.3)	0.9 (0.7–1.3)
Others	2.3 (1.4–3.8)*	1.8 (1.02–3.2)*	1.7 (0.9–2.8)
SGA			
Well-nourished (reference)			
Moderately-severely malnourished	2.0 (1.6–2.6)*	1.4 (1.02–1.8)*	1.4 (1.1–1.9)*
ECOG score			
0–1 (reference)			
2–4	1.6 (1.2–2.1)*	1.2 (0.9–1.6)	
Season of diagnosis			
Summer (reference)			
Fall	1.0 (0.7–1.4)	1.4 (0.9–1.9)	
Winter	1.2 (0.9–1.7)	1.3 (0.9–1.8)	
Spring	1.1 (0.8–1.5)	1.1 (0.8–1.6)	
Smoking status			
Never (reference)			
Former	1.5 (1.1–2.1)*	1.4 (1.01–2.1)*	1.5 (1.1–2.2)*
Current	1.6 (1.1–2.1)*	1.3 (0.9–1.9)	1.4 (0.9–1.9)
Age (continuous)	1.0 (0.99–1.01)	1.01 (0.99–1.02)	
Serum vitamin D (continuous)	1.0 (0.99–1.01)		
BMI (continuous)	0.97 (0.95–0.99)*	0.99 (0.97–1.02)	
Serum albumin (continuous)	0.44 (0.35–0.54)*	0.46 (0.34–0.61)*	0.46 (0.35–0.61)*

(*SGA* Subjective Global Assessment, *ECOG* Eastern Cooperative Oncology Group, *CTCA* Cancer Treatment Centers of America, *PA* Pennsylvania, *IL* Illinois, *OK* Oklahoma, *BMI* Body Mass Index, *ngcpaml* nanograms per milliliter, *HR* Hazard Ratio, *CI* Confidence Interval)

*P < = 0.05

To account for potential sampling bias and further investigate the stability of the classical multivariate Cox model reported in Table 3, we conducted a bootstrap resampling procedure based on 1000 samples. We did not find any significant differences in regression coefficients and their corresponding p values between the classical Cox regression and bootstrap Cox regression models.

## Discussion

We investigated the association between serum 25(OH)D and survival in newly diagnosed stages III-IV NSCLC patients. The findings of our study add to the growing body of literature on the potential association between serum vitamin D and survival in NSCLC.

Consistent with the findings published by Heist et al. [21], Anic et al. [23] and Turner et al. [22] we did not find a significant association between serum vitamin D and survival in our cohort of newly diagnosed advanced NSCLC patients. The lack of a significant association between serum vitamin D and survival in our study could be explained in several ways. First, the disease was far too advanced in our patients for vitamin D levels to have any impact on prognosis. Second, the vitamin D levels in our study were perhaps too low to have any significant impact on the prognosis. Lastly, vitamin D may not have any true impact on survival in advanced NSCLC. Collectively, the results of our study considered against the backdrop of the existing literature in this area suggest that serum vitamin D levels measured either pre- or post-diagnosis might not be independently predictive of survival in advanced NSCLC cancer after controlling for the most Season of diagnosis, ECOG performance status, smoking status and hospital location were found to be statistically significantly associated with serum vitamin D levels. Patients diagnosed in the summer and fall months were less likely to be deficient in vitamin D compared to those diagnosed in winter and spring, a finding that has been widely reported in the literature. However, the mean serum vitamin D levels across all 4 seasons were less than 32 ng/ml, a level considered to be sufficient [12, 14]. As a result, consistent with the previous literature [18], the patients in our cohort were not exposed to enough sunlight even during the summer months, and therefore had low circulating 25(OH)D levels. Patients with good performance status were less likely to be deficient in vitamin D compared to those with poor performance status. This finding is not surprising because patients with good performance status can be assumed to be more physically active compared to those with poor performance status. We found that current smokers had a greater prevalence of vitamin D deficiency compared to past or no smokers. By contrast, the study by Anic et al. did not report an association between smoking status and serum vitamin D [23]. There is little information in the literature on the potential biologic mechanisms underlying the relationship between smoking status and serum vitamin D levels. However, given the findings of our study, smoking status is clearly an important covariate to include in all studies evaluating the role of serum vitamin D in predicting mortality in all tobacco-related cancers such as NSCLC. Finally, patients diagnosed at our Philadelphia hospital had a lower prevalence of vitamin D deficiency compared to patients diagnosed at Zion and Tulsa hospitals. This could potentially be attributed to referral bias or might reflect geographic variation in serum vitamin D distribution.

Two predictors demonstrated borderline significance in their association with serum vitamin D levels: serum albumin and nutritional status. Patients with vitamin D deficiency had lower serum albumin levels compared to those with non-deficient serum vitamin D levels, and well-nourished patients had a lower prevalence of vitamin D deficiency compared to malnourished patients; both findings consistent with our recently reported research in prostate cancer [17].

In contrast with previously published research [13, 28–30], we did not find lower serum levels of 25(OH)D to be associated with higher BMI. Multiple mechanisms have been proposed to explain the association of obesity with hypovitaminosis D, including lack of sunlight exposure from physical inactivity [31] and sequestration of vitamin D in subcutaneous fat depots [32] As a result, it has been proposed that BMI should be taken into account when assessing a patient’s vitamin D status and more aggressive vitamin D supplementation should be considered in obese cancer patients [30]. This lack of association between serum vitamin D and BMI in our study could be due to a lack of significant variability in BMI levels given our patients’ advanced disease status.

In vitro and animal studies have shown that vitamin D has antiproliferative, antimetastasis, and antiangiogenesis activities in lung cancer and may modulate the immune function of lung epithelial cells [18, 33–35]. In human squamous cell carcinoma and lung cancer cell lines and mouse models, 1,25-dihydroxyvitamin D has been shown to inhibit the growth and angiogenesis of tumor cells potentially due to the suppression of response to vascular endothelial growth factor [35]. Also, 1, 25-dihydroxyvitamin D suppresses epidermal growth factor receptor, which signals several tumorigenic processes, such as proliferation and metastasis, in lung cancer [36]. Finally, 1, 25-dihydroxyvitamin D has been hypothesized to stimulate the secretion of protein glues, such as E-cadherin and catenin, making cells more adherent to each other thereby preventing metastases [37].

There are some limitations of our study that are worth acknowledging. This is an association study which cannot prove causality. Reverse causality (the effect of cancer on serum vitamin D levels) is always a possibility in observational studies and cannot be ruled out with certainty [38]. Potential confounding by factors such as exercise, sunlight exposure and dietary vitamin D intake cannot be ruled out, although season of diagnosis was used as a proxy for sunlight exposure. In this study, serum 25(OH)D was measured only once at the time of diagnosis which might not be reflective of vitamin D levels during cancer generation or progression. However, previous research has shown the reliability of a single serum vitamin D assessment over a 5-year period [28]. Similarly, smoking status was only available at baseline based on the information provided by patients. The treatments received were not standardized as they would have been in a clinical trial setting. No formal sample size calculation was conducted before undertaking this study. Finally, the available survival data could not distinguish between death from NSCLC and from other causes; therefore, we assessed the all-cause mortality instead of NSCLC-specific mortality.

There are some strengths of our study. We examined a homogeneous patient population of newly diagnosed advanced NSCLC which minimizes potential confounding by tumor stage and prior treatment history. We measured serum vitamin D at disease diagnosis prior to receiving any treatment which eliminates the possibility of treatment and lifestyle changes affecting serum vitamin D levels after diagnosis. We had a large sample size of histologically confirmed NSCLC cases. By using a consecutive case series of all eligible patients seen at our institution during a fixed time period, we minimized the possibility of selection bias in our study. Lastly, we adjusted for a wide range of potential clinical and demographic confounders thereby minimizing the possibility of residual confounding. That being said, the possibility of residual confounding can never be completely ruled out in observational studies.

## Conclusion

In conclusion, we did not find any significant association between serum 25(OH)D and survival in newly diagnosed stages III-IV NSCLC patients. This finding needs further exploration in future prospective studies of larger sample sizes across all stages of NSCLC.

## Abbreviations

25(OH)D: 25-hydroxyvitamin D
NSCLC: non-small-cell lung cancer
SGA: subjective global assessment
1,25(OH)_2_D: 1,25-dihydroxyvitamin D
CTCA: Cancer Treatment Centers of America
IRB: Institutional Review Board
CLIA: chemiluminescence immune assay
RIA: radioimmunoassay
BMI: body mass index
HRs: hazard ratios
CIs: confidence intervals
PH: proportional hazards
BCa: bias-corrected and accelerated
ECOG: Eastern Cooperative Oncology Group
ng/ml: nanograms per milliliter
SD: standard deviation
g/dl: grams per deciliter
IBM: International Business Machines
SPSS: statistical package for social sciences
kg/m2: kilograms per meter squared
